# News and Miscellany

**Published:** 1903-06

**Authors:** 


### News and Miscellany.

---

Married.—At Austin, Texas, June 3rd, by Rev. R. J. Briggs, Dr. F. E. Daniel and Miss Josie Draper. No cards.

---

Rare Chance for Doctor.—Residence and $3000 practice; railroad town, county seat. Little opposition. Also, drug store and stock of drugs. Address "West Texas," care Texas Medical Journal.

---

Will Repair Your Electrical Machines.—Static and all Electrical Medical Apparatus put in running order. I am also agent for electrical and X-ray apparatus. Oliver Brush, 710 Colorado Street, Austin, Texas.

EXAMINATION of urine, sputum, blood, pathological specimens, etc., made at reasonable rates by New Orleans Clinical Laboratory, 124 Barrone Street, New Orleans. O. L. Pothier, M. D., Charity Hospital. I. I. Lemann, M. D., Secretary. J. B. Guthrie, M. D.

---

A REFERENCE CHART of the Diseases of Nervous System and Muscles, by Edward Curtis Hill, M. Sc., M. D. See advertisement of this chart by Antikamnia Chemical Company, in front form of this issue. A copy of the chart can be had free by asking for it; mentioning the *Red Back.*

---

THE PASSING OF THE BEARD.—The beard is rapidly going out of fashion. It is tabooed in New York by the swell set, and the Emperor of Germany has issued orders that every army, navy and hospital surgeon, assistant surgeon, nurse and employe shall be clean shaved every day, and that they shall use exclusively the *Shumate Dollar Razor.* See advertisement.

---

THE VENERABLE AND BELOVED Dr. Samuel Hollingsworth Stout, now 81 years of age, the immortal medical director of all the hospitals in the Southern Confederacy—has removed from Dallas to Clarendon, Donley county, Texas. I am glad to note that he is still in the enjoyment of excellent health physically and mentally. Long may he live to enjoy the repose and tranquillity to which his long and efficient services to his country and the afflicted richly entitle him. He is still writing the History of the Hopsital Department of the Southern Confederacy, which is running serially through the *Southern Practitioner,* Nashville, the official organ of the United Confederate Veterans Association. This, when completed, will be issued in book form. It will be a monument to his name and service "more enduring than brass."

---

DR. CORNELL.—If there is a doctor in Texas who does not know the genial and delightful Cornell—the ubiquitous Cornell—he ought to be ashamed to let it be known. Cornell is the detail man of Sharp & Dohme, the Texas representative, and he is as well known and *nearly* as popular as their standard and staple products, chief among which is their elegant pharmaceutical *"Glycerophosphates."* It is the very highest type of the tonic and reconstructive. I've tried it, and I know whereof I speak. Cornell was in to see me the other day, and left me feeling good. I was like little

Tommy Grace who had a pain in his face so bad he couldn't learn a letter. In came Dickey Long, singing such a funny little song that Tommy laughed, and his face got better. Substitute Cornell for Dickey Long, and funny little joke for funny little song, and you've got it. Cornell is a warm number; the most popular salesman in Texas. Long may he "sale."

